# Some Observations on Ptyalism, with Cases

**Published:** 1815-06

**Authors:** W. Robertson


*For the Medical and Physical Journal.*

### *Some Observations on Ptyalism, with Cases;* by W. ROBERTSON, M. D. A.O.L. and C.C.E.

"Sans doute mucus animale a ses movements, sa surabondance, ou sòn exces, ses metastases, ses trausports lents ou subits sur d'outres parties. Examineé dans celle nouvelle face, l'humeur muqueuse doit interesser les medicins, &c.*"—*Annales de Mus.* vol. xiii. p. 61. Paris, 1808.

SAUVAGES (Genus 261), Linnè (177), Vogel (103), Sagar (197), and Cullen (120), each take notice of this disease in their respective Nosologies, and the latter observes, "Si quis ptyalismus idiopathicus sit pro eo habere vellem, ptyalismum a laxitate." S. sp. 4, tom. 2, p. 345.

Ptyalism, as a symptom of disease, is by no means a rare occurrence, and, as a consequence of the introduction of mercury into the system, is a well known affection; but the subsequent cases do not arrange themselves under either of these heads, being referable to an idiopathic disease. The quantity of saliva, afforded by the salivary glands in a state of health, is very considerable, as is known from practical observations;† and this secretion appears to be essentially requisite to the well-being of the animal economy, not only in the performance of mastication, but in the process of digestion; for, when this fluid is ejected from the mouth, instead of being permitted to pass into the stomach, dyspepsia, hypochondriasis, and a variety of chronic evils are the invariable consequences. It is of importance also to observe, that, when the system has once put on the condition of an increased secretion, whether from disease or the pernicious use of stimulants and narcotics, it is with some difficulty that the habit

---

* Dr. Bostock, in his excellent disquisition on Animal Substances, (Edinburgh Medical and Surgical Journal, vol. i.) considers mucus as constituting one half of the solid contents of saliva. See Fourcroy and Vauquelin on Animal Mucus, p. 61, vol. xiii. Dr. Pearson, Philosophical Transactions, 1809. Fordyce, Thesaur. Med. vol. ii. Bichat, Traité des Membranes, p. 32.

† Vide Academ. des Sciences, 1719, mem. p. 452.

habit of determination to the salival glands can be obviated, even though the causes by which it may have been primarily induced, are withdrawn.* A knowledge of this circumstance, conjoined to the certainty that the continuance of the ptyalism, by impairing the function of digestion, and inducing a general relaxation of the system, exerts a powerful influence in prolonging the disease itself, should operate as a cogent motive for combating the affection as soon as it becomes apparent.

In the first of the subsequent cases, the disease was of short duration, though accompanied by the characteristic symptoms of laxity of fibre and sallowness of complexion, which were so predominant in the second case, of two years continuance; yet none of the most powerful medicines of the tonic class seemed to have any influence, either in permanently obviating the general laxity of the system, or in diminishing the inordinate determination to the glands of the fauces. The disease ran its course, till, apparently checked by atmospheric influence, it gradually subsided.

The other two cases appeared to yield immediately to the remedy prescribed for their cure, and have not re-appeared. The disease, in one of the three cases, was attributed, by the party herself, to the use of mercury, though that medicine had not been exhibited for two years previously.

It will be remarked, that these cases occurred in the autumnal months, or were so much aggravated at that season, as to require medical aid; a season in which there exists an evident relaxation and repressure, as well in the animal as vegetable kingdoms.† It will, no doubt, be noted also, that the disease appeared at periods of life when considerable changes are wont to occur in the human system.‡

CASE

---

* See an interesting case in the Medical Transactions, as related by Sir George Baker, in which the ptyalism continued apparently from habit, after the cause (a mechanical one) had been removed.

† This liability or predisposition to disease in autumn is noticed by most of the older writers, who attended much more to this subject than modern physicians do. Celsus, in particular, observes, "per autumnum vero, propter cœli varietatem, periculum maximum est." "Quo fit ut autumnus plurimos opprimat. nam fere meridianis temporibus calor: nocturnis atque matutenis, simulque etiam vespertinis, frigus est. Corpus ergo & æstate, & subinde meridianis caloribus relaxatum, subito frigore excipitur."—De Medicina, cap. 3, lib. 1, & cap. 5, lib. 2.

‡ The subject of the first case had arrived at that period, when the catamenia, for the most part, take their final leave, though there

Case I.—September 5, 1814. I was requested to visit Mrs. ———, ætatis 44, who complained that she laboured under a constant overflow of saliva, which not unfrequently passed involuntarily over the lip in a stream, an occurrence which was particularly obnoxious to her, and disgusting to those with whom inclination or necessity induced her to associate. The quantity excreted in the twenty-four hours might be two pints, but there was considerable variation in this respect, which variation, however, was not referable to any known cause or law which she could assign. On some days it did not exceed a pint; on others it amounted to three times this quantity. She found her strength (both corporeal and mental) much impaired at the expiration of a month's continuance of this disease, and, within a few days, she had become oppressed with flatulence, low spirits, and irregularity of the bowels. She felt, to use her own phraseology, "relaxed all over." She also remarked, that on one or two occasions, she had swallowed the superabundant saliva, but so much nausea (which terminated in a diarrhœa) had been induced from this cause, that she had resolved not to repeat the experiment. In other respects her health was much as it was wont to be. It appears, that, two years ago, she was under the necessity of undergoing a course of mercury, for the cure of a local disease, to such an extent as to induce ptyalism, and to have that condition kept up for some weeks. She conceives that her present disease (which commenced a month ago) is a consequence of the course of mercury she had previously undergone.

On inspection, the gums appear pale and flaccid, and there is no perceptible fœtor. The body indicates much flaccidity, conjoined to corpulency. The face is pale, puffed up, and sallow; the lips little tinged from the blood's colour,

---

there had not occurred any symptom indicative of that event; on the contrary, the patient menstruated regularly. The second case was that of a youth of fourteen years of age, whose voice was of the neuter gender, and whose masculine form was only about to be evolved. The third instance of this disease, was a female, who had arrived at that æra when the body undergoes many changes from age alone.

We have also witnessed this disease as a precursor of a first fit of gout, though the latter did not seem to influence the progress of the former. See Heberden (de destillatione) Comment. p. 121, who, in writing of this disease (ptyalism), observes, "Podagra supervenit sine profecter." The gout, for the most part, "appears at a certain period of life when the system is liable to a loss of tone."—See Cullen's Pathology of this disease, vol. ii. p. 85.

and and the mind appears to labour under dejection, listlessness, and apathy. Pulse 60. Skin cold and clammy. Catamenia regular, both in relation to time and quantity. She has never been subject to hysteria.*

℞. Aloes Socotrinæ, Pulveris Rhabarbari āa ʒiſs.

Extracti Gentianæ, ʒſs.

Syrupi Zingiberis q. s. fiat massa in pilulas l. dividenda, quarum sumat. 3 pro dosi 3tia quaque nocte.

℞. Decocti Cinchonæ, ℔. i.

Tincturæ Muriatis Ferri, ʒij.

M. Capiat, ℥iſs. ter in die. Descendat in balneum Aquæ frigidæ summo mane indies.

℞. Corticis Quercus concisi, ʒij. Aquæ, ℥xij. coque ad colaturæ, ℥viij. & adde Aluminis Sulphatis, ʒi. Syrupi Rosæ, ʒſs. Utatur pro Gargarismate, in horas vel sœpius.

12th. Has used the medicines assiduously. The quantity of saliva has somewhat diminished, and her strength is improved. Continuentur Remedia.

19th. Little amendment. Continuentur Medicamenta.

26th. Ptyalism continues with little or no abatement. Omittantur Decoctum Cinchonæ & Pilulæ. Sumat Vini Aloes ʒſs. omni nocte & mane.

October 3d. Ptyalism much diminished, which she attributes to a frequent exposure to the atmosphere, which is now cold.

10th. Ptyalism gone; is in her usual health.

Case II.—November 6th. I was consulted by the parents of Mr. ——, a youth of fourteen years of age. Two years ago, he was attacked by a contagious fever, which confined

* See Heberden Comment. (de destillatione) who observes, " menstruis inordinatis, inter alias valetudinis perturbationes subsecutum est immodicum salivæ profluvium; quod etiam mulieres hystericæ expertæ sunt in duos tresve menses, perinde ac si vivo argento delebutæ fuissent," p. 120. I, however, experience some doubts in conceiving a ptyalism of three years' duration, being induced by a moderate use of mercury alone. Heberden seems to attach doubt to the propriety, in many instances, of suppressing this affection, and hesitates whether it should be designated a disease or a remedy; but surely a malady of this nature, which, from habit, (so powerful an agent in the animal economy) is apt to become permanent, and is so disgusting in itself, independent of its consequences, should be suppressed as soon as may be, in every instance.

him three weeks to the house; during his convalescence, he experienced a constant inclination to spit, so much so that he could not sleep from the unremitting flow of saliva which wetted his pillow, and rendered him otherwise most uncomfortable. This discharge has continued ever since, decreasing, however, very considerably during the warm months of the year, and attended by a proportionate augmentation in winter. He conceives that the great excretion of perspirable matter which uniformly takes place from his skin during summer, and the suppression thereof in winter which occurs as regularly, may be considered as the causes of the alternations above-mentioned. Having never used any receptacle to contain the saliva, he, consequently, has formed no determinate idea of the quantity excreted, but says, that it is very great. He is of a slender form and sallow complexion. The surface appears greasy, and his hands are cool and clammy. Functions natural.

℞. Pulveris Jalapij ℥i.

Gummi Gambogiæ gr. xv.

Olej. Menthæ gtt. iij.

Syrupi q. s. fiant Pilulæ xv. Sumat 1 pro dosi 3tia quaque nocte.

℞. Oxidi Bismuthi Albi gr. v.
Pulveris Gummi Tragacanthæ gr. xv.
M. Sumat ter in die ex quovis vehiculo crasso.

13th. Ptyalism much reduced in quantity. Continuentur remedia.

20th. Spitting has ceased. Omittantur Medicamenta.*

Case III.—December 2d. My advice was requested in favour of Miss ———, æt. 65, who, for some weeks past, has been afflicted with ptyalism, which commenced without any apparent cause. She is confined to her bed-room, where I found her sitting with the usual chamber bason, nearly one half full of saliva, which she averred to have flowed from her mouth in the course of the morning, and that the amount in the day reached from two to three pints

* The oxyd of bismuth was recommended by Dr. Marcet as a remedy in gastrodynia.—See Memoirs of the London Medical Society, vol. v. And also by Dr. Bradsley, Medical Reports, p. 218. But it is to Dr. Odier, of Geneva, that we are indebted for the introduction of this valuable oxyd. We have found it a medicine of unequalled efficacy in many affections of the stomach, attended by a general want of vigour. Its eminent quality in relieving and curing gastric diseases, attended by this condition of the system, first suggested to us its use in ptyalism.—See Journal de Medicine par Corvisart, vol. xiii. p. 280.

generally.

generally. She expresssed the utmost abhorrence of the disease, and conceived that its commutation for extreme pain would be desirable. She observed, that, with the exception of occasional attacks of stomach complaints, she had enjoyed excellent health. Pulse 65, and of good strength. Visage sallow, and eyes a little tinged from bile. Skin cold, clammy, and flaccid. Has never been subject to hysteria, nor has she, to the best of her knowledge, ever used mercury. Bowels irregular. Urine high coloured.

℞. Submuriatis Hydrargyri, gr. v.
Pulveris Jalapij, gr. x.
Olej Menthæ, gtt. ij. Sumat statim sub forma boli pro cathartico.
Capiat Vini Aloes, ℥i. 3tia quaque nocte. Applicentur hora somni Cataplasmata ex semine synapeos, pedibus, more solito.

℞. Oxidi Bismuthi Albi, gr. x.
Pulveris gummi Tragacanthæ, gr. xxv. M. Sumat ter in die, ex Syrupo.

9th. Feet still sore from the synapisms. Ptyalism considerably diminished, one half at least. Continuentur remedia.

17th. Spitting nearly gone. Continuentur medicamenta.

24th. The ptyalism has ceased. Omittantur medicamenta. Capiat Infusi Cinchonæ, ℥iſs. ter in die cum Tincturæ Muriatis Ferri, gtt. xv. singulis dosibus.

31st. Is free from complaint.

---

*For the Medical and Physical Journal.*

